# The efficacy of resveratrol supplementation on inflammation and oxidative stress in type-2 diabetes mellitus patients: randomized double-blind placebo meta-analysis

**DOI:** 10.3389/fendo.2024.1463027

**Published:** 2025-01-13

**Authors:** Peiye Zhu, Yunrui Jin, Jiya Sun, Xia Zhou

**Affiliations:** ^1^ College of Pharmacy and Bioengineering, Chongqing University of Technology, Chongqing, China; ^2^ Department of Rehabilitation, Chongqing Orthopedic Hospital of Traditional Chinese Medicine, Chongqing, China; ^3^ Department of Acupuncture and Moxibustion, Jiading Hospital of Traditional Chinese Medicine, Shanghai, China; ^4^ Department of Traditional Chinese Medicine, Zigong First People’s Hospital, Zigong, Sichuan, China

**Keywords:** resveratrol, inflammation, antioxidant, meta-analysis, type-2 diabetes mellitus

## Abstract

**Background:**

The effects of resveratrol supplementation on inflammation and oxidative stress in patients with type 2 diabetes mellitus (T2DM) were controversial. A meta-analysis was performed to assess the changes in levels of inflammation and oxidative stress in patients with T2DM.

**Methods:**

Relevant literatures before November 6, 2024 were screened through Web of Science,Embase,the Cochrane Library and other sources (ClinicalTrials, ProQuest Dissertations and Theses). The quality of the literature was evaluated according to the Cochrane Handbook of Systematic Reviews. The study quality was assessed using the risk-of-bias 2 tool and the Grading of Recommendations Assessment,Development and Evaluation (GRADE) system. Review Manager 5.3 conducted meta-analysis of the data included in the literature.

**Results:**

This meta-analysis was conducted in six randomized controlled trials involving 533 participants. Our results showed that supplementation with resveratrol significantly reduced C-reactive protein levels(SMD = -1.40, 95%CI(-2.60, -0.21), P = 0.02; Level of evidence: low), lipid peroxide levels (SMD = -0.99, 95%CI(-1.36, -0.61), P < 0.00001; Level of evidence: low), 8-isoprostanes(SMD = -0.79, 95%CI(-1.16, -0.42), P < 0.0001; Level of evidence: low) and oxidative stress score (SMD = -1.62, 95%CI(-2.49, -0.75), P = 0.0003; Level of evidence: very low). In addition, compared to placebo, Supplementation with resveratrol significantly increased glutathione peroxidase levels (SMD = 0.38, 95%CI(0.03, 0.74), P = 0.04; Level of evidence:low) and catalase levels (SMD = 0.33, 95%CI(0.03, 0.63), P = 0.03; Level of evidence: low). However, no significant difference was observed in improving interleukin-6 levels (SMD = -1.35, 95%CI(-2.75, -0.05), P = 0.06; Level of evidence: very low), tumor necrosis factor α levels (SMD = -3.30, 95%CI(-7.47, 0.87), P = 0.12; Level of evidence: very low), superoxide dismutase levels (SMD = 0.39, 95%CI(-0.26, 1.04), P = 0.24; Level of evidence: very low), total antioxidant capacity levels (SMD = 0.39, 95%CI(-0.23, 1.00), P = 0.21; Level of evidence: very low) and malondialdehyde levels (SMD = -3.36, 95%CI(-10.30, 3.09), P = 0.29; Level of evidence: very low).

**Conclusion:**

Resveratrol improved inflammation and oxidative stress in T2DM patients to some extent. This provides a new idea and method for clinical treatment. However, due to the limitations of the study, more large-sample, multi-center clinical studies are needed to verify this conclusion.

## Introduction

Diabetes mellitus (DM), a metabolic disease characterized by chronic hyperglycemia, has become an epidemic worldwide (1). The International Diabetes Federation (IDF) reports that the global prevalence of DM among people aged 20-79 is expected to be 10.5% (536.6 million cases) in 2021, rising to 12.2%(783.2 million cases) by 2045 (2). Type 2 diabetes mellitus (T2DM) is a disease characterized by high blood sugar symptoms caused by islet dysfunction and cell resistance to insulin (3, 4). Chronic high blood sugar can lead to serious complications, including kidney disease, neuropathy and retinopathy, as well as microangiopathy and large vascular disease, which can seriously affect patients’ quality of life (5–8).

At present, the treatment of T2DM mainly includes drug therapy, diet control and exercise therapy (9–12). However, the current treatment plan still has some shortcomings, such as drug treatment may bring some side effects, diet control and exercise therapy need patients to adhere to for a long time, and the effect is limited. T2DM patients often have difficulty changing their eating habits, so they can easily turn to dietary supplements to control the disease (13, 14).

Active substances from plants, including curcumin, pipeline, resveratrol, and carotene, are essential for health (15–19). Supplementation of these active substances can reduce the risk of cardiovascular disease, neurodegenerative diseases, T2DM, etc. (20–24). These supplements are not only designed to have anti-hyperglycemic effects, but also to reduce inflammatory responses and oxidative stress to prevent DM complications (1, 25, 26). Resveratrol is a natural polyphenolic compound found in grapes, peanuts and knotweed (27, 28). Recent studies have found that resveratrol has anti-inflammatory, antioxidant, hypoglycemic and other pharmacological effects (29). T2DM is a chronic metabolic disease in which patients are prone to inflammation and oxidative stress (30, 31). Resveratrol has strong antioxidant and anti-inflammatory effects, and can regulate the inflammatory response and oxidative stress level in the body through various ways, so it is expected to be an effective treatment option for T2DM patients (32). Therefore, it is of great significance to study the effects of resveratrol on inflammation and oxidative stress in T2DM patients.

In recent years, some studies in animal models of diabetes have shown that resveratrol supplementation can reduce inflammation and oxidative stress (33). However, clinical trials have shown controversial results (34–39). The effects of resveratrol supplementation on inflammation and oxidative stress in T2DM patients were unknown. The aim of this study was to investigate the effects of resveratrol on inflammation and oxidative stress in T2DM patients through a randomized double-blind placebo meta-analysis.

## Methods

### Search strategy

We searched Pubmed, Web of Science,Embase,the Cochrane Library and other sources (ClinicalTrials, ProQuest Dissertations and Theses) for randomized controlled trials (RCTs) on the effects of resveratrol on inflammation and oxidative stress in patients withT2DM published from the beginning of the database to November 6, 2024. Use the following search terms: resveratrol, type-2 diabetes mellitus, randomized controlled trial, randomized, etc. Search strategies and search results for each database can be found in the **Supplementary Materials**.

### Including and excluding criteria

#### Inclusion criteria

(1) Participants: Patients diagnosed with T2DM;
(2) Interventions: Oral resveratrol. The dosage and frequency of supplementation were not limited;
(3) Controls: The placebo was similar to the intervention group;
(4) Outcomes: Outcomes associated with oxidative stress and inflammatory response;
(5) Study design: Randomized controlled trial.

#### Exclusion criteria

(1) Animal experiments;
(2) The data in the article was not reliable;
(3) The original data cannot be extracted, and the full text of the literature cannot be obtained.

### Data extraction

Two researchers performed literature screening, data extraction and cross-checking independently. In case of disagreement, discuss with the third party to resolve. The contents to be extracted include: (1) basic information included in the study; (2) Basic characteristics of population; (3) Details of interventions; (4) Key points for assessing the risk of bias; (5) Outcome data.

### Risk of bias

The risk of bias of RCTs will be assessed using Cochrane risk of bias (RoB) 2 tool. The evaluation included 6 items: 1) the bias in the randomization process; 2) Bias away from established interventions; 3) Bias in outcome measurement; 4) Bias due to missing outcome data; 5) Bias in selective reporting of results; 6) Overall bias.

### Certainty of the evidence

We will assess the certain of evidence using the Grading of Recommendations Assessment, Development and Evaluation (GRADE) tool. Since the original studies included were all RCTs, the evidence quality level was initially high, but it would be downgraded due to the risk of bias, inconsistency, incoherence, inaccuracy, and publication bias of the original study. The final quality of evidence was divided into four levels: “high”, “moderate”, “low” and “very low”.

### Data synthesis and statistical analysis

Meta-analysis was performed using RevMan 5.3 software. Continuous variables were expressed using standard mean difference (SMD) and its corresponding 95% confidence interval (CI). Statistical heterogeneity of test analysis: If P ≤ 0.1 and I(2) > 50%, it indicated that there was a large heterogeneity among the test results, and the random effects model was used for pooled analysis. On the contrary, the fixed effect model was used. Subgroup analysis was performed according to the dose of resveratrol. The stability of the meta-analysis results was verified by sensitivity analysis using the replacement effect model. The funnel plot was used to determine whether there was publication bias.

## Results

### Characteristics of included studies

**Figure 1:**
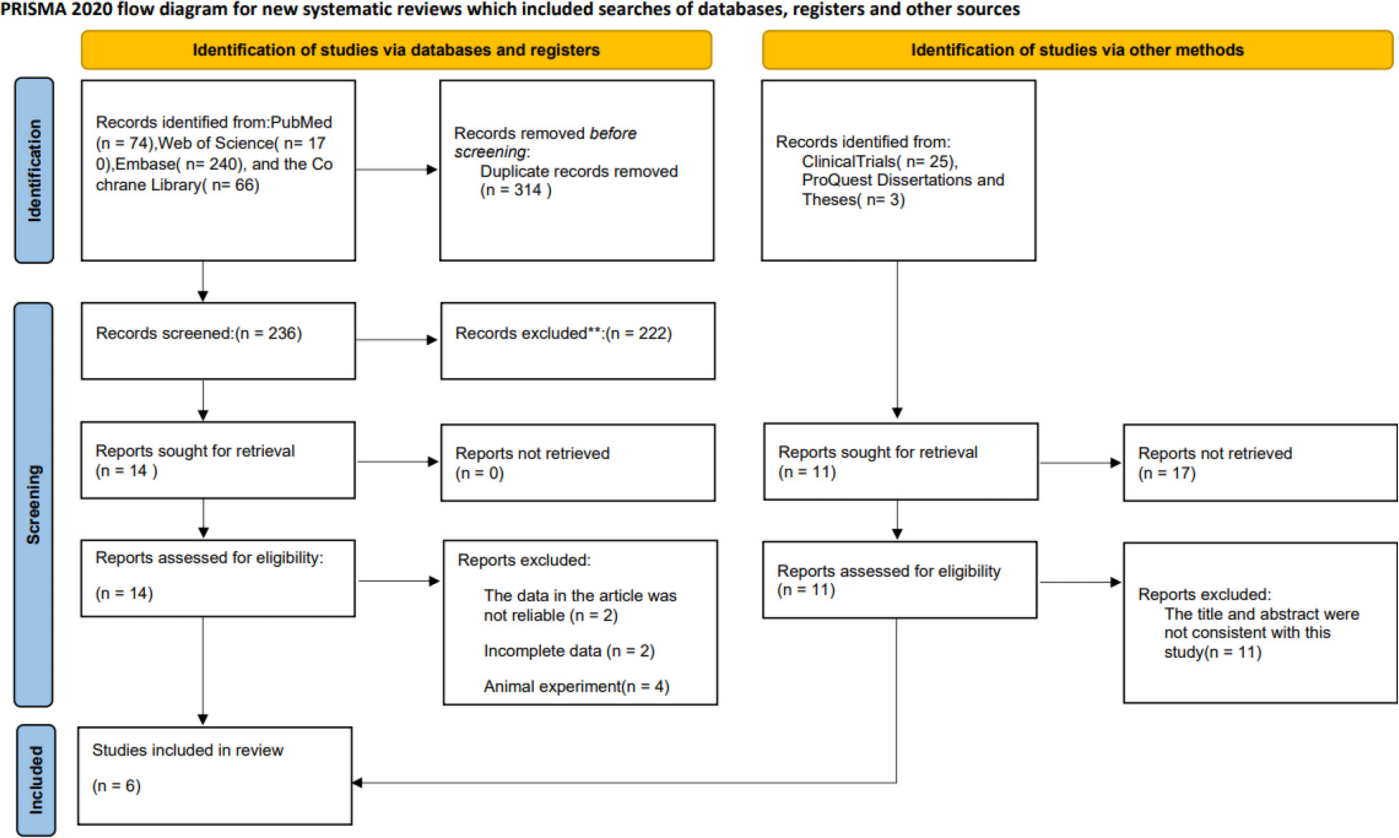
Flow diagram of study selection.

**Figure 2:**
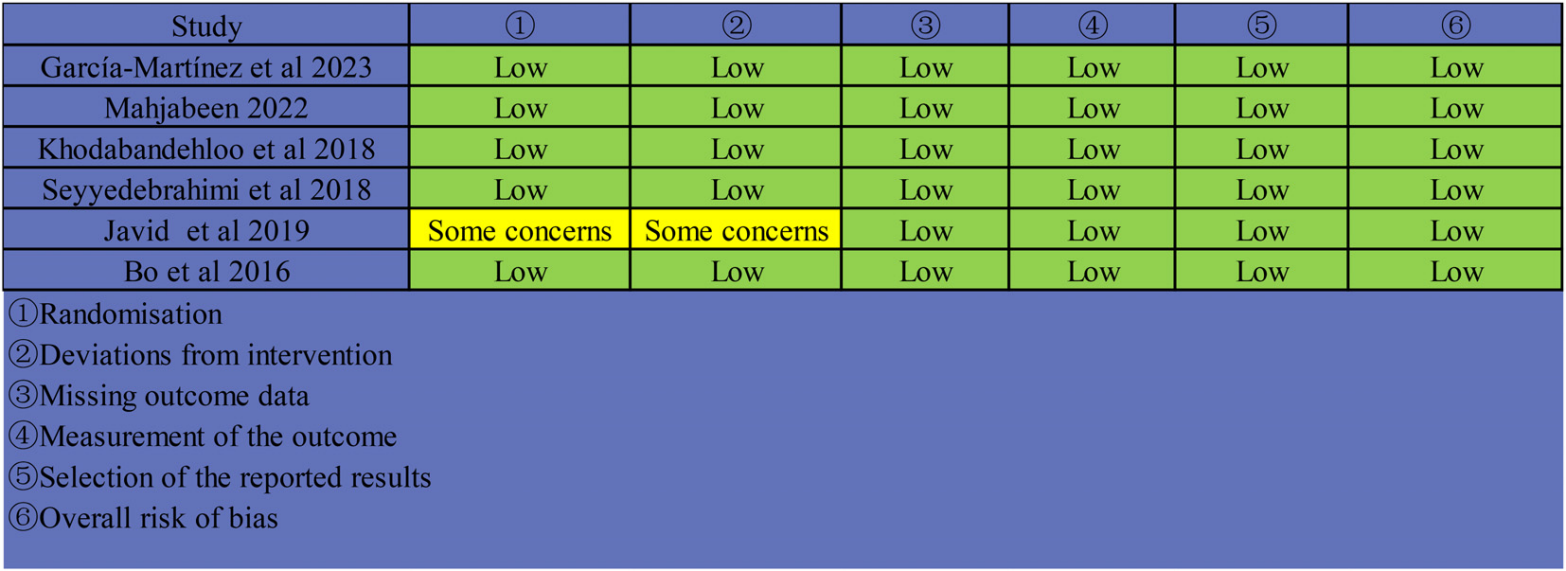
Risk of bias assessment by cochrane risk-of-bias tool for included RCTs.

Through searching the database and other resources, a total of 578 articles were retrieved. 314 duplicates were excluded using Endnote literature management software. After reading the title and abstract, 237 articles were excluded. After reading the full text, eight literatures were excluded according to the inclusion criteria and exclusion criteria, and six (34–39) literatures were finally included(**Figure 1**). All trials were registered. There were two three-arm trials (34, 35), so we split them into two arm trials for analysis. One trial (35)was conducted in Mexico. One trial (38) was conducted in Pakistan. One trial (34) was conducted in Italy. The remaining three trials (36, 37, 39) were conducted in Iran. The dose of Resveratrol ranges from 40mg to 1000mg. The duration of intervention ranged from 4 to 24 weeks. The basic characteristics of the included literatures were shown in **Table 1**. The included RCTs were of high quality (**Figure 2**).

**Table 1:** Characteristics of the included randomized controlled trials.

Study, year	Country	Registration number	Participants		Ages		Intervention		Dose	Duration
			Treatment group	Control group	Treatment group	Control group	Treatment group	Control group		
García-Martínez et al., 2023	Mexico	ISRCTN15172592	37	28	66 ± 6	64 ± 5	Resveratrol	Placebo	1000 mg	24 weeks
			32		64 ± 7		Resveratrol		500 mg	
Mahjabeen 2022	Pakistan	SLCTR/2018/019	55	55	49.42 ± 9.04	50.02 ± 12.57	Resveratrol	Placebo	200 mg	24 weeks
Khodabandehloo et al., 2018	Iran	IRCT2015080223336N2	25	20	56.48 ± 6.72	61.10 ± 5.61	Resveratrol	Placebo	800 mg	12 weeks
Seyyedebrahimi et al., 2018	Iran	IRCT2015072523336N1	23	23	54.96 ± 6.37	58.72 ± 6.06	Resveratrol	Placebo	800 mg	8 weeks
Javid et al., 2019	Iran	IRCT2015012420765N1	21	22	30-60		Resveratrol	Placebo	480 mg	4 weeks
Bo et al., 2016	Italy	NCT02244879	65	62	65.0 ± 7.6	65.4 ± 8.8	Resveratrol	Placebo	500 mg	24 weeks
			65		64.9 ± 8.6		Resveratrol		40 mg	

### C-reactive protein

**Figure 3:**
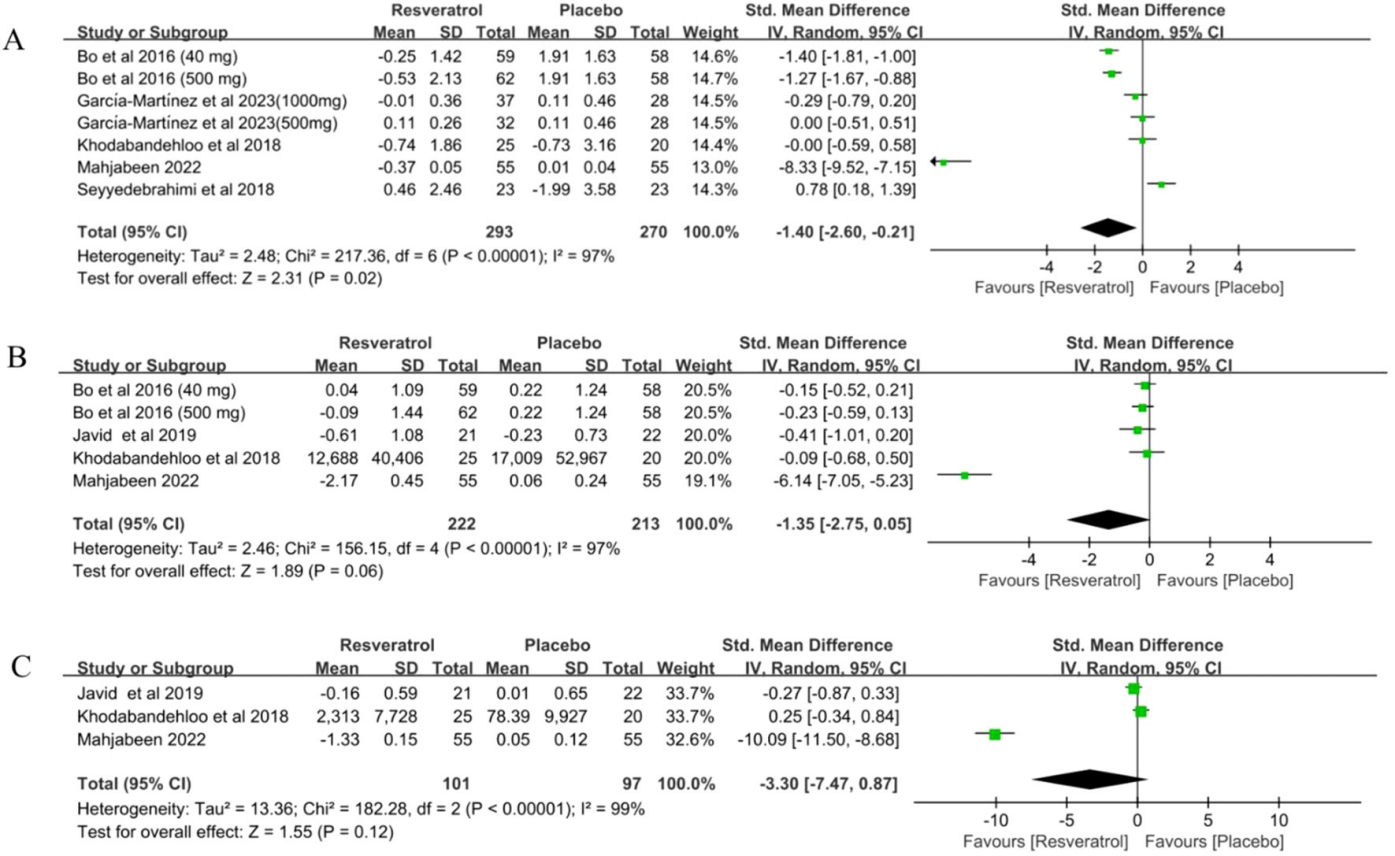
Forest plot for effect of resveratrol supplementation on biomarkers of inflammation **(A)** C-reactive protein, **(B)** interleukin-6 and **(C)** tumor necrosis factor α in type-2 diabetes mellitus patients.

Seven studies (34–39) involving 563 participants reported C-reactive protein(CRP) levels in patients with T2DM before and after treatment. The results of the meta-analysis showed that resveratrol significantly reduced CRP levels compared with placebo (SMD = -1.40, 95%CI(-2.60, -0.21), P = 0.02) (**Table 2**, **Figure 3A**). Sensitivity analysis was conducted by fixed effect model. Meta-analysis of this outcome were stable (SMD = -0.81, 95%CI(-1.00, -0.62), P < 0.00001)(**Table 2**).

**Table 2:** Sensitivity analysis of each outcomes.

Outcomes	Pooled analysis results			Change model analysis results		
	Model	SMD(95%CI)	*P*-value	Model	SMD(95%CI)	*P*-value
C-reactive protein	Random	**-1.40(-2.60, -0.21)**	**0.02**	Fixed	**-0.81(-1.00, -0.62)**	**P < 0.00001**
Interleukin-6	Random	-1.35(-2.75, 0.05)	0.06	Fixed	**-0.53(-0.74, -0.32)**	**P < 0.00001**
Tumor necrosis factor α	Random	-3.30(-7.47, 0.87)	0.12	Fixed	**-0.83(-1.24, -0.43)**	**P < 0.0001**
Lipid peroxide	Fixed	**-0.99(-1.36, -0.61)**	**P < 0.00001**	Random	**-0.99(-1.36, -0.61)**	**P < 0.00001**
8- isoprostanes	Fixed	**-0.79(-1.16, -.042)**	**P < 0.0001**	Random	**-0.79(-1.29, -0.29)**	**0.002**
Superoxide dismutase	Random	0.39(-0.26, 1.04)	0.24	Fixed	**0.43(0.12, 0.74)**	**0.006**
Glutathione peroxidase	Fixed	**0.38(0.03, 0.74)**	**0.04**	Random	**0.38(0.03, 0.74)**	**0.04**
Catalase	Fixed	**0.33(0.03, 0.63)**	**0.03**	Random	**0.33(0.03, 0.63)**	**0.03**
Total antioxidantcapacity	Random	0.39(-0.23, 1.00)	0.21	Fixed	**0.40(0.09, 0.71)**	**0.01**
Oxidative stress score	Random	**-1.62(-2.49, -0.75)**	**0.0003**	Fixed	**-1.58(-1.99, -1.17)**	**P < 0.00001**
Malondialdehyde	Random	-3.60(-10.30, 3.09)	0.29	Fixed	**-1.87(-2.38, -1.37)**	**P < 0.00001**

SMD, Standard mean difference; CI, Confidence interval; Bold characters indicate statistically significant differences.

Bold font indicates that the difference is statistically significant.

### Interleukin-6

Five studies (34, 36–38) involving 435 participants reported interleukin-6 (IL-6) levels in patients with T2DM before and after treatment. The results of the meta-analysis showed that resveratrol did not reduce IL-6 levels compared to placebo (SMD = -1.35, 95%CI(-2.75, -0.05), P = 0.06) (**Table 2**, **Figure 3B**). Sensitivity analysis was conducted by fixed effect model. The meta-analysis of this outcome was reversed (SMD = -0.53, 95%CI(-0.74, -0.32), P < 0.00001)(**Table 2**). This suggests that resveratrol may significantly reduce IL-6 levels in T2DM patients (**Table 2**).

### Tumor necrosis factor α

Three studies (36–38) involving 198 subjects reported tumor factor α(TNF-α) levels in patients with T2DM before and after treatment. The results of the meta-analysis showed that resveratrol did not significantly reduce TNF-α levels compared to placebo (SMD = -3.30, 95%CI(-7.47, 0.87), P = 0.12) (**Table 2**, **Figure 3C**). Sensitivity analysis was conducted by fixed effect model. The meta-analysis of this outcome was reversed(SMD = -0.83, 95%CI(-1.24, -0.43), P < 0.0001). This suggests that resveratrol may significantly reduce TNF-α levels in T2DM patients (**Table 2**).

### Lipid peroxide

**Figure 4:**
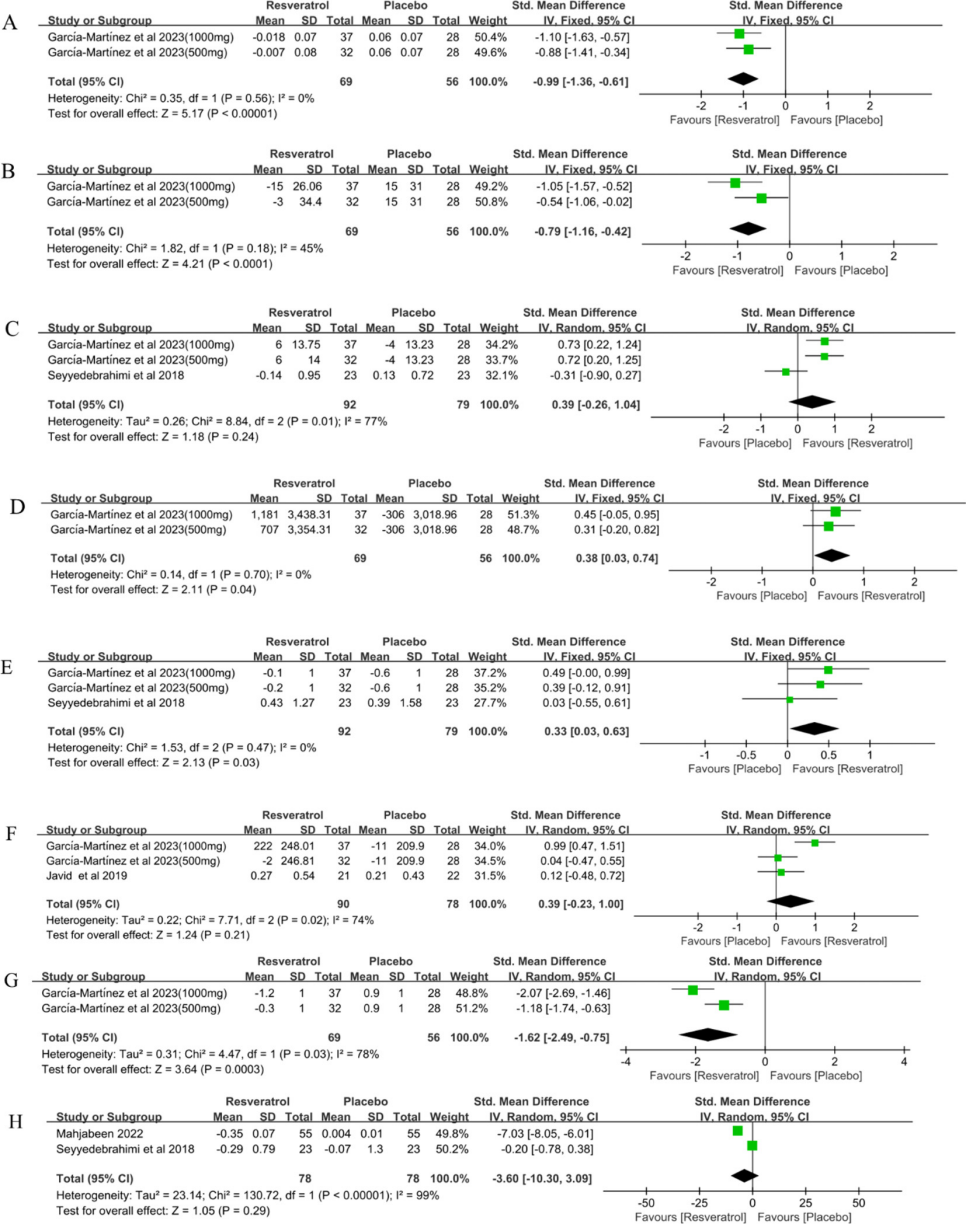
Forest plot for effect of resveratrol supplementation on oxidative stress **(A)** lipid peroxide, **(B)** 8-isoprostanes, **(C)** superoxide dismutase, **(D)** glutathione peroxidase, **(E)** catalase, **(F)** total antioxidant capacity, **(G)** oxidative stress score and **(H)** malondialdehyde in type-2 diabetes mellitus patients.

Two studies (35) involving 125 participants reported lipid peroxide(LPO) levels in patients with T2DM before and after treatment. The results of the meta-analysis showed that resveratrol significantly reduced LPO levels compared to placebo(SMD = -0.99, 95%CI(-1.36, -0.61), P < 0.00001) (**Table 2**, **Figure 4A**). Random effects model was used for sensitivity analysis. Meta-analyses of this outcome were stable (SMD = -0.99, 95%CI(-1.36, -0.61), P < 0.00001)(**Table 2**).

### 8-isoprostanes

Two studies (35) involving 125 participants reported 8-isoprostanes levels in patients with T2DM before and after treatment. The results of the meta-analysis showed that resveratrol significantly reduced 8-isoprostanes levels compared with placebo (SMD = -0.79, 95%CI(-1.16, -0.42), P < 0.0001) (**Table 2**, **Figure 4B**). Random effects model was used for sensitivity analysis. Meta-analyses of this outcome were stable (SMD = -0.79, 95%CI(-1.29, -0.29), P = 0.002)(**Table 2**).

### Superoxide dismutase

Three studies (35, 39) involving 171 participants reported superoxide dismutase (SOD) levels in patients with T2DM before and after treatment. The results of the meta-analysis showed that resveratrol did not significantly increase SOD levels compared with placebo (SMD = 0.39, 95%CI(-0.26, 1.04), P = 0.24) (**Table 2**, **Figure 4C**). Sensitivity analysis was conducted by fixed effect model. The meta-analysis of this outcome was reversed (SMD = 0.43, 95%CI(0.12, 0.74), P = 0.006). This suggests that resveratrol may significantly increase SOD levels in T2DM patients (**Table 2**).

### Glutathione peroxidase

Two studies (35) involving 125 participants reported glutathione peroxidase (GPx) levels in patients with T2DM before and after treatment. The results of the meta-analysis showed that resveratrol significantly increased GPx levels compared to placebo (SMD = 0.38, 95%CI(0.03, 0.74), P = 0.04) (**Table 2**, **Figure 4D**). Random effects model was used for sensitivity analysis. Meta-analyses of this outcome were stable (SMD = 0.38, 95%CI(0.03, 0.74), P = 0.04)(**Table 2**).

### Catalase

Three studies (35, 39) involving 171 participants reported catalase (Cat) levels in patients with T2DM before and after treatment. The results of the meta-analysis showed that resveratrol significantly increased Cat levels compared to placebo (SMD = 0.33, 95%CI(0.03, 0.63), P = 0.03) (**Table 2**, **Figure 4E**). Random effects model was used for sensitivity analysis. Meta-analyses of this outcome were stable (SMD = 0.33, 95%CI(0.03, 0.63), P = 0.03)(**Table 2**).

### Total antioxidant capacity

Three studies (35, 36) involving 168 participants reported Total antioxidant capacity(TAC) levels before and after treatment in patients with T2DM. The results of the meta-analysis showed that resveratrol did not significantly increase TAC levels compared to placebo(SMD = 0.39, 95%CI(-0.23, 1.00), P = 0.21) (**Table 2**, **Figure 4F**). Sensitivity analysis was conducted by fixed effect model. The meta-analysis of this outcome was reversed (SMD = 0.40, 95%CI(0.09, 0.71), P = 0.01). This suggests that resveratrol may significantly increase TAC levels in patients with T2DM (**Table 2**).

### Oxidative stress score

Two studies (35) involving 125 subjects reported oxidative stress scores (OSS) in patients with T2DM before and after treatment. The results of the meta-analysis showed that resveratrol significantly reduced OSS compared to placebo(SMD = -1.62, 95%CI(-2.49, -0.75), P = 0.0003) (**Table 2**, **Figure 4G**). Sensitivity analysis was conducted by fixed effect model. Meta-analyses of this outcome were stable (SMD = -1.58, 95%CI(-1.99, -1.17), P < 0.00001)(**Table 2**).

### Malondialdehyde

Two studies (38, 39) involving 156 participants reported malondialdehyde (MDA) levels in patients with T2DM before and after treatment. The results of the meta-analysis showed that resveratrol did not significantly reduce MDA levels compared to placebo(SMD = -3.36, 95%CI(-10.30, 3.09), P = 0.29) (**Table 2**, **Figure 4H**). Sensitivity analysis was conducted by fixed effect model. The meta-analysis of this outcome was reversed (SMD = -1.87, 95%CI(-2.38, -1.37), P < 0.00001). This suggests that resveratrol may significantly reduce MDA levels in T2DM patients (**Table 2**).

### Adverse event

All studies investigated the occurrence of adverse events. The results showed that resveratrol had a high safety profile with no adverse events.

### Subgroup analysis

We performed subgroup analyses of CRP and IL-6 based on the dose of resveratrol (< 500mg vs ≥ 500mg). The results showed that no difference was observed whether the dose of resveratrol was < 500mg or ≥ 500mg (P > 0.05)(**Table 3**).

**Table 3:** Subgroup analysis.

Outcomes	Dose	No. of studies	Heterogeneity		SMD(95%CI)	*P* Value
			I2	P		
C-reactive protein	< 500 mg	2	99.2%	< 0.0001	-4.88(-11.72, 1.96)	0.162
	≥ 500mg	5	89.7%	< 0.0001	-0.18(-0.88, 0.53)	0.626
Interleukin-6	< 500 mg	3	99%	< 0.0001	-2.20(-5.12, 0.71)	0.140
	≥ 500mg	2	0	0.7	-0.19(-0.50, 0.12)	0.220

SMD, Standard mean difference; CI, Confidence interval

### Publication bias analysis

**Figure 5:**
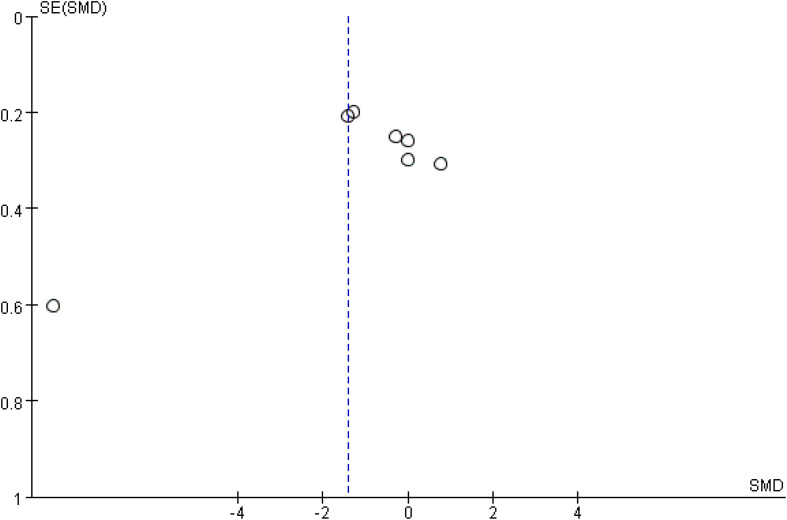
Funnel diagram of C-reactive protein.

We conducted a publication bias analysis for CRP. The results showed that the funnel plot was asymmetrical and there may be publication bias (**Figure 5**).

### Evidence quality evaluation

The quality of evidence for each outcome measure was assessed using the GRADE system. RCTs without major defects are by default the highest level of evidence in GRADE. The quality of the evidence was evaluated and processed according to 5 downgrade factors and 3 upgrade factors. The evidence quality of 5 outcome indicators was low, and the rest was very low (**Table 4**).

**Table 4:** GRADE systematic evaluation of evidence quality.

Outcomes	Quality assessment					Intervention/Control	SMD(95%CI)	Quality of evidence
	Risk of bias	Inconsistency	Indirectness	Imprecision	Publication bias			
C-reactive protein	Not serious	Serious^2^	Not serious	Not serious	Serious^4^	293/270(7 studies)	-1.40(-2.60, -0.21)	Low: ⨁⨁◯◯
Interleukin-6	Limitations^1^	Serious^2^	Not serious	Not serious	Serious^5^	222/213(5 studies)	-1.35(-2.75, 0.05)	Very low: ⨁◯◯◯
Tumor necrosis factor α	Limitations^1^	Serious^2^	Not serious	Serious^3^	Serious^5^	101/97(3 studies)	-3.30(-7.47, 0.87)	Very low: ⨁◯◯◯
Lipid peroxide	Not serious	Not serious	Not serious	Serious^3^	Serious^5^	69/56(2 studies)	-0.99(-1.36, -0.61)	Low: ⨁⨁◯◯
8- isoprostanes	Not serious	Not serious	Not serious	Serious^3^	Serious^5^	69/56(2 studies)	-0.79(-1.16, -.042)	Low: ⨁⨁◯◯
Superoxide dismutase	Not serious	Serious^2^	Not serious	Serious^3^	Serious^5^	92/97(3 studies)	0.39(-0.26, 1.04)	Very low: ⨁◯◯◯
Glutathione peroxidase	Not serious	Not serious	Not serious	Serious^3^	Serious^5^	69/56(2 studies)	0.38(0.03, 0.74)	Low: ⨁⨁◯◯
Catalase	Not serious	Not serious	Not serious	Serious^3^	Serious^5^	92/97(3 studies)	0.33(0.03, 0.63)	Low: ⨁⨁◯◯
Total antioxidantcapacity	Limitations^1^	Serious^2^	Not serious	Serious^3^	Serious^5^	90/78(3 studies)	0.39(-0.23, 1.00)	Very low: ⨁◯◯◯
Oxidative stress score	Not serious	Serious^2^	Not serious	Serious^3^	Serious^5^	69/56(2 studies)	-1.62(-2.49, -0.75)	Very low: ⨁◯◯◯
Malondialdehyde	Not serious	Serious^2^	Not serious	Serious^3^	Serious^5^	78/78(2 studies)	-3.60(-10.30, 3.09)	Very low: ⨁⨁◯◯

^1^Some RCTs did not mention using the randomized scheme hiding or randomized grouping; ^2^Significant heterogeneity;^3^The study sample size was small;^4^ The funnel plot was asymmetrical; ^5^The number of studies was small.

## Discussion

This meta-analysis is the most comprehensive data available on the effects of resveratrol supplementation on inflammation and oxidative stress in patients with T2DM. Many previous meta-analyses have confirmed that resveratrol can improve insulin resistance in T2DM patients, reduce fasting blood glucose and insulin levels, improve cardiometabolic parameters, and improve blood lipids (29, 40–43). Resveratrol is a polyphenol compound found naturally in grape skins, red wine and other foods, and is widely studied and believed to have anti-inflammatory and antioxidant effects (44). DM is a common chronic metabolic disease, often accompanied by inflammation and oxidative stress (45). In studies conducted in animal models of T2DM, resveratrol has been shown to have antioxidant, anti-inflammatory and even hypoglycemic effects (33).

This meta-analysis evaluated the overall effect of resveratrol on inflammation and oxidative stress in patients with T2DM by summarizing the results of six RTCs, involving 533 participants. In this meta-analysis, we found that resveratrol supplementation had a modest effect on inflammation and oxidative stress levels in patients with T2DM, particularly in reducing CRP levels, LPO levels, 8-isoprostanes levels, and OSS. At the same time, we observed that resveratrol supplementation increased GPx levels and Cat levels, further confirming its antioxidant effects. However, no significant differences were observed on some measures, such as IL-6, TNF-α, SOD, TAC, and MDA levels. Although our meta-analysis found no significant differences observed on certain markers of inflammation and oxidative stress, such as IL-6, TNF-α, SOD, TAC, and MDA levels. However, when we performed sensitivity analysis, we found that these outcomes were statistically significant. Due to the small number of studies included in this meta-analysis and the limited sample size, no significant differences in these outcomes were observed. The evidence quality of the relevant outcome indicators of the included literatures in this study is very low, which may affect the reliability of the research conclusions.

Resveratrol can reduce inflammation in T2DM patients in a variety of ways. The inflammatory response in T2DM patients is closely associated with hyperglycemia, leading to insulin resistance and impaired islet beta cell function (46, 47). Studies have shown that resveratrol can inhibit the activation of NF-κB signaling pathway and reduce the release of inflammatory factors such as TNF-α and IL-6, thereby reducing inflammatory response and improving insulin sensitivity, thus helping to control blood sugar levels in T2DM patients (35). In addition, resveratrol can inhibit inflammation by regulating toll-like receptor signaling pathways and cytokine signaling pathways associated with inflammation (48). Secondly, resveratrol can also reduce oxidative stress in T2DM patients through antioxidant effects (46). The hyperglycemic state of T2DM patients leads to excessive production of free radicals in the body, which exceeds the clearance capacity of the antioxidant system, thus triggering oxidative stress response and impairs cell structure and function (49, 50). Studies have shown that resveratrol can increase the activity of antioxidant enzymes and reduce the level of oxidative stress indicators such as MDA, thereby reducing the damage caused by oxidative stress (48). These effects are mediated by several intracellular signaling pathways, including nuclear factor κB inhibitor kinase/nuclear factor (NF) κB inhibitor/NF-κB pathway, adenosine phosphate kinase pathway, phosphatidylinositol-3 kinase/protein kinase B/endothelial nitric oxide synthase, etc (35, 46, 51). Resveratrol can improve insulin resistance by upregulating miRNA mmu-miR-363-3p through PI3K-Akt pathway and prevent pancreatic β cell damage and dysfunction (52, 53). Resveratrol can restore pancreatic β cells by inhibiting p38/p16MAPK pathway through SIRT1-dependent pathway, thus effectively improving ethanol-induced diabetes (54, 55). Resveratrol activates SIRT-1/NF-κB signaling pathway to reduce cellular inflammation and oxidative stress (56, 57). The anti-inflammatory effect of Resveratrol is mainly achieved by reducing cellular inflammation and oxidative stress by regulating STAT1 and SIRT1 signaling pathways. Studies have shown that Resveratrol inhibits the expression of COX-2 and iNOS by blocking the activation of NF-κB (58). In addition, Resveratrol can regulate the expression of NF-κB/Nrf 2 after H2O2 treatment (59). Therefore, part of the efficacy of Resveratrol, including anti-inflammatory antioxidant effects, may be mediated by the NF-κB/Nrf-2 pathway. In general, resveratrol can reduce the level of inflammation and oxidative stress in T2DM patients by inhibiting the release of inflammatory factors and enhancing antioxidant capacity, thus playing a certain role in improving the condition of DM. Future studies can further explore the potential mechanism of resveratrol in the treatment of diabetes and provide more references and guidance for clinical treatment.

## Limitation

First of all, because there were few RCT trials in this field, the sample size was insufficient, which affected the research results. Second, the dose and intervention time of resveratrol included in the study were different, which also affected the evaluation effect of this study. Third, most of the included documents come from Middle Eastern countries, which may have ethnic and regional differences. Finally, due to limitations in the number of included studies and the type of specific intervention, we did not conduct more subgroup analyses. Therefore, we suggest that readers should take these limitations into account when applying the conclusions of this study.

## Conclusion

Resveratrol improved inflammation and oxidative stress in T2DM patients to some extent. The relevant mechanism may be related to its antioxidant and anti-inflammatory effects, which has certain guiding significance for clinical practice. However, due to the limitations of the study, more large-sample, multi-center clinical studies are needed to verify this conclusion, so as to better guide clinical practice.

## Data Availability

The datasets presented in this study can be found in online repositories. The names of the repository/repositories and accession number(s) can be found in the article/**Supplementary Material**.

## References

[1] Darenskaya M Kolesnikov S Semenova N Kolesnikova L . Diabetic nephropathy: significance of determining oxidative stress and opportunities for antioxidant therapies. Int J Mol Sci. (2023) 24:12378. doi: 10.3390/ijms241512378 37569752 PMC10419189

[2] Sun H Saeedi P Karuranga S Pinkepank M Ogurtsova K Duncan BB . IDF Diabetes Atlas: Global, regional and country-level diabetes prevalence estimates for 2021 and projections for 2045. Diabetes Res Clin practice. (2022) 183:109119. doi: 10.1016/j.diabres.2021.109119 PMC11057359 34879977

[3] Dong C Liu R Li R Huang Z Sun S . Effects of traditional chinese exercises on glycemic control in patients with type 2 diabetes mellitus: A systematic review and meta-analysis of randomized controlled trials. . Sports Med (Auckland NZ). (2024) 54:2327–55. doi: 10.1007/s40279-024-02166-2 38874898

[4] Lin X Zhu K Qiu Z Li R Li L Lu Q . Associations between beverage consumption and risk of microvascular complications among individuals with type 2 diabetes. J Clin Endocrinol Metab. (2024) 12:dgae242. doi: 10.1210/clinem/dgae242 38687598

[5] Chen M Pu L Gan Y Wang X Kong L Guo M . The association between variability of risk factors and complications in type 2 diabetes mellitus: a retrospective study. Sci Rep. (2024) 14:6357. doi: 10.1038/s41598-024-56777-w 38491155 PMC10943073

[6] Faselis C Katsimardou A Imprialos K Deligkaris P Kallistratos M Dimitriadis K . Microvascular complications of type 2 diabetes mellitus. Curr Vasc Pharmacol. (2020) 18:117–24. doi: 10.2174/1570161117666190502103733 31057114

[7] Pradhan D Sahu PK Purohit S Ranajit SK Acharya B Sangam S . Therapeutic interventions for diabetes mellitus-associated complications. Curr Diabetes Rev. (2024). doi: 10.2174/0115733998291870240408043837 38706367

[8] Yang DR Wang MY Zhang CL Wang Y . Endothelial dysfunction in vascular complications of diabetes: a comprehensive review of mechanisms and implications. Front endocrinology. (2024) 15:1359255. doi: 10.3389/fendo.2024.1359255 PMC11026568 38645427

[9] Garza MC Pérez-Calahorra S Rodrigo-Carbó C Sánchez-Calavera MA Jarauta E Mateo-Gallego R . Effect of aromatic herbs and spices present in the mediterranean diet on the glycemic profile in type 2 diabetes subjects: A systematic review and meta-analysis. Nutrients. (2024) 16:756. doi: 10.3390/nu16060756 38542668 PMC10975382

[10] Al-Mhanna SB Rocha-Rodriguesc S Mohamed M Batrakoulis A Aldhahi MI Afolabi HA . Effects of combined aerobic exercise and diet on cardiometabolic health in patients with obesity and type 2 diabetes: a systematic review and meta-analysis. BMC sports science Med rehabilitation. (2023) 15:165. doi: 10.1186/s13102-023-00766-5 PMC10696788 38049873

[11] Dyńka D Kowalcze K Ambrozkiewicz F Paziewska A . Effect of the ketogenic diet on the prophylaxis and treatment of diabetes mellitus: A review of the meta-analyses and clinical trials. Nutrients. (2023) 15:500. doi: 10.3390/nu15030500 36771207 PMC9919384

[12] Sánchez-Rosales AI Guadarrama-López AL Gaona-Valle LS Martínez-Carrillo BE Valdés-Ramos R . The effect of dietary patterns on inflammatory biomarkers in adults with type 2 diabetes mellitus: A systematic review and meta-analysis of randomized controlled trials. Nutrients. (2022) 14:4577. doi: 10.3390/nu14214577 36364839 PMC9654560

[13] Watanabe M Risi R Masi D Caputi A Balena A Rossini G . Current evidence to propose different food supplements for weight loss: A comprehensive review. Nutrients. (2020) 12:2873. doi: 10.3390/nu12092873 32962190 PMC7551574

[14] Hannon BA Fairfield WD Adams B Kyle T Crow M Thomas DM . Use and abuse of dietary supplements in persons with diabetes. Nutr diabetes. (2020) 10:14. doi: 10.1038/s41387-020-0117-6 32341338 PMC7186221

[15] Gu Y Niu Q Zhang Q Zhao Y . Ameliorative effects of curcumin on type 2 diabetes mellitus. Molecules (Basel Switzerland). (2024) 29:2934. doi: 10.3390/molecules29122934 38930998 PMC11206386

[16] Widjanarko ND Tamio E Jusni LFJ Alvianto S Arifin ES Iryaningrum MR . Effects of combination of curcumin and piperine supplementation on glycemic profile in patients with prediabetes and type 2 diabetes mellitus: A systematic review and meta-analysis. J ASEAN Fed Endocrine Societies. (2024) 39:106–14. doi: 10.15605/jafes.039.01.18 PMC11163317 38863920

[17] Gimblet CJ Kruse NT Geasland K Michelson J Sun M Mandukhail SR . Effect of resveratrol on endothelial function in patients with CKD and diabetes: A randomized controlled trial. Clin J Am Soc Nephrology: CJASN. (2023) 19:161–8. doi: 10.2215/CJN.0000000000000337 PMC10861109 37843843

[18] Fathalipour M Fathalipour H Safa O Nowrouzi-Sohrabi P Mirkhani H Hassanipour S . The therapeutic role of carotenoids in diabetic retinopathy: A systematic review. Diabetes Metab syndrome obesity: Targets Ther. (2020) 13:2347–58. doi: 10.2147/DMSO.S255783 PMC7342496 32753919

[19] Arabshomali A Bazzazzadehgan S Mahdi F Shariat-Madar Z . Potential benefits of antioxidant phytochemicals in type 2 diabetes. Molecules (Basel Switzerland). (2023) 28:7209. doi: 10.3390/molecules28207209 37894687 PMC10609456

[20] Vitale M Masulli M Rivellese AA Bonora E Cappellini F Nicolucci A . Dietary intake and major food sources of polyphenols in people with type 2 diabetes: The TOSCA. IT Study. Eur J Nutr. (2018) 57:679–88. doi: 10.1007/s00394-016-1355-1 28004268

[21] Bozkurt O Kocaadam-Bozkurt B Yildiran H . Effects of curcumin, a bioactive component of turmeric, on type 2 diabetes mellitus and its complications: an updated review. Food Funct. (2022) 13:11999–2010. doi: 10.1039/D2FO02625B 36367124

[22] Panahi Y Hosseini MS Khalili N Naimi E Majeed M Sahebkar A . Antioxidant and anti-inflammatory effects of curcuminoid-piperine combination in subjects with metabolic syndrome: A randomized controlled trial and an updated meta-analysis. Clin Nutr (Edinburgh Scotland). (2015) 34:1101–8. doi: 10.1016/j.clnu.2014.12.019 25618800

[23] Mohammadipoor N Shafiee F Rostami A Kahrizi MS Soleimanpour H Ghodsi M . Resveratrol supplementation efficiently improves endothelial health: A systematic review and meta-analysis of randomized controlled trials. Phytotherapy research: PTR. (2022) 36:3529–39. doi: 10.1002/ptr.v36.9 35833325

[24] Muscolo A Mariateresa O Giulio T Mariateresa R . Oxidative stress: the role of antioxidant phytochemicals in the prevention and treatment of diseases. Int J Mol Sci. (2024) 25:3264. doi: 10.3390/ijms25063264 38542238 PMC10970659

[25] Panahi Y Khalili N Sahebi E Namazi S Simental-Mendía LE Majeed M . Effects of curcuminoids plus piperine on glycemic, hepatic and inflammatory biomarkers in patients with type 2 diabetes mellitus: A randomized double-blind placebo-controlled trial. Drug Res. (2018) 68:403–9. doi: 10.1055/s-0044-101752 29458218

[26] Tshivhase AM Matsha T Raghubeer S . The protective role of resveratrol against high glucose-induced oxidative stress and apoptosis in HepG2 cells. Food Sci Nutr. (2024) 12:3574–84. doi: 10.1002/fsn3.v12.5 PMC11077230 38726423

[27] Molani-Gol R Rafraf M . Effects of resveratrol on the anthropometric indices and inflammatory markers: an umbrella meta-analysis. Eur J Nutr. (2024) 63:1023–40. doi: 10.1007/s00394-024-03335-9 38374352

[28] Zeraattalab-Motlagh S Jayedi A Shab-Bidar S . The effects of resveratrol supplementation in patients with type 2 diabetes, metabolic syndrome, and nonalcoholic fatty liver disease: an umbrella review of meta-analyses of randomized controlled trials. Am J Clin Nutr. (2021) 114:1675–85. doi: 10.1093/ajcn/nqab250 34320173

[29] Delpino FM Figueiredo LM . Resveratrol supplementation and type 2 diabetes: a systematic review and meta-analysis. Crit Rev Food Sci Nutr. (2022) 62:4465–80. doi: 10.1080/10408398.2021.1875980 33480264

[30] Klisic A Karakasis P Patoulias D Khalaji A Ninić A . Are oxidative stress biomarkers reliable part of multimarker panel in female patients with type 2 diabetes mellitus. Metab syndrome related Disord. (2024) 22:679–85. doi: 10.1089/met.2024.0100 38848276

[31] Yang Z Zhang L Liu J Li D . Litchi pericarp extract treats type 2 diabetes mellitus by regulating oxidative stress, inflammatory response, and energy metabolism. Antioxidants (Basel Switzerland). (2024) 13:495. doi: 10.3390/antiox13040495 38671942 PMC11047702

[32] Maleki V Foroumandi E Hajizadeh-Sharafabad F Kheirouri S Alizadeh M . The effect of resveratrol on advanced glycation end products in diabetes mellitus: a systematic review. Arch Physiol Biochem. (2022) 128:253–60. doi: 10.1080/13813455.2019.1673434 32125189

[33] Hu HC Lei YH Zhang WH Luo XQ . Antioxidant and anti-inflammatory properties of resveratrol in diabetic nephropathy: A systematic review and meta-analysis of animal studies. Front Pharmacol. (2022) 13:841818. doi: 10.3389/fphar.2022.841818 35355720 PMC8959544

[34] Bo S Ponzo V Ciccone G Evangelista A Saba F Goitre I . Six months of resveratrol supplementation has no measurable effect in type 2 diabetic patients. A randomized double blind placebo-controlled trial. Pharmacol Res. (2016) 111:896–905. doi: 10.1016/j.phrs.2016.08.010 27520400

[35] García-Martínez BI Ruiz-Ramos M Pedraza-Chaverri J Santiago-Osorio E Mendoza-Núñez VM . Effect of resveratrol on markers of oxidative stress and sirtuin 1 in elderly adults with type 2 diabetes. Int J Mol Sci. (2023) 24:1–15. doi: 10.3390/ijms24087422 PMC10138491 37108584

[36] Javid AZ Hormoznejad R Yousefimanesh HA Haghighi-Zadeh MH Zakerkish M . Impact of resveratrol supplementation on inflammatory, antioxidant, and periodontal markers in type 2 diabetic patients with chronic periodontitis. Diabetes Metab syndrome. (2019) 13:2769–74. doi: 10.1016/j.dsx.2019.07.042 31405706

[37] Khodabandehloo H Seyyedebrahimi S Esfahani EN Razi F Meshkani R . Resveratrol supplementation decreases blood glucose without changing the circulating CD14(+)CD16(+) monocytes and inflammatory cytokines in patients with type 2 diabetes: a randomized, double-blind, placebo-controlled study. Nutr Res (New York NY). (2018) 54:40–51. doi: 10.1016/j.nutres.2018.03.015 29914666

[38] Mahjabeen W Khan DA Mirza SA . Role of resveratrol supplementation in regulation of glucose hemostasis, inflammation and oxidative stress in patients with diabetes mellitus type 2: A randomized, placebo-controlled trial. Complementary therapies Med. (2022) 66:102819. doi: 10.1016/j.ctim.2022.102819 35240291

[39] Seyyedebrahimi S Khodabandehloo H Nasli Esfahani E Meshkani R . The effects of resveratrol on markers of oxidative stress in patients with type 2 diabetes: a randomized, double-blind, placebo-controlled clinical trial. Acta diabetologica. (2018) 55:341–53. doi: 10.1007/s00592-017-1098-3 29357033

[40] García-Martínez BI Ruiz-Ramos M Pedraza-Chaverri J Santiago-Osorio E Mendoza-Núñez VM . Influence of age and dose on the effect of resveratrol for glycemic control in type 2 diabetes mellitus: systematic review and meta-analysis. Molecules (Basel Switzerland). (2022) 27:5232. doi: 10.3390/molecules27165232 36014469 PMC9416262

[41] Abdelhaleem IA Brakat AM Adayel HM Asla MM Rizk MA Aboalfetoh AY . The effects of resveratrol on glycemic control and cardiometabolic parameters in patients with T2DM: A systematic review and meta-analysis. Medicina clinica. (2022) 158:576–85. doi: 10.1016/j.medcli.2021.06.028 34666902

[42] Zhang T He Q Liu Y Chen Z Hu H . Efficacy and safety of resveratrol supplements on blood lipid and blood glucose control in patients with type 2 diabetes: A systematic review and meta-analysis of randomized controlled trials. Evid Based Complement Alternat Med. (2021) 2021:5644171. doi: 10.1155/2021/5644171 34484395 PMC8410426

[43] Nyambuya TM Nkambule BB Mazibuko-Mbeje SE Mxinwa V Mokgalaboni K Orlando P . A meta-analysis of the impact of resveratrol supplementation on markers of renal function and blood pressure in type 2 diabetic patients on hypoglycemic therapy. Molecules (Basel Switzerland). (2020) 25:5645. doi: 10.3390/molecules25235645 33266114 PMC7730696

[44] Esfahani M Rahbar AH Asl SS Bashirian S Mir Moeini ES Mehri F . The effects of resveratrol on silica-induced lung oxidative stress and inflammation in rat. Saf Health at work. (2023) 14:118–23. doi: 10.1016/j.shaw.2023.02.001 PMC10024237 36941929

[45] Ghavidel F Amiri H Tabrizi MH Alidadi S Hosseini H Sahebkar A . The combinational effect of inulin and resveratrol on the oxidative stress and inflammation level in a rat model of diabetic nephropathy. Curr developments Nutr. (2024) 8:102059. doi: 10.1016/j.cdnut.2023.102059 PMC10826146 38292928

[46] Banaszak M Górna I Woźniak D Przysławski J Drzymała-Czyż S . The impact of curcumin, resveratrol, and cinnamon on modulating oxidative stress and antioxidant activity in type 2 diabetes: moving beyond an anti-hyperglycaemic evaluation. Antioxidants (Basel Switzerland). (2024) 13:510. doi: 10.3390/antiox13050510 38790615 PMC11117755

[47] Zuo X Yao R Zhao L Zhang Y Lu B Pang Z . Campanumoea javanica Bl. activates the PI3K/AKT/mTOR signaling pathway and reduces sarcopenia in a T2DM rat model. Acupuncture and Herbal Medicine. (2022) 2:99–108. doi: 10.1097/hm9.0000000000000027

[48] Koushki M Farahani M Yekta RF Frazizadeh N Bahari P Parsamanesh N . Potential role of resveratrol in prevention and therapy of diabetic complications: a critical review. Food Nutr Res. (2024) 68:9731. doi: 10.29219/fnr.v68.9731 PMC11075469 38716357

[49] Dludla PV Mabhida SE Ziqubu K Nkambule BB Mazibuko-Mbeje SE Hanser S . Pancreatic β-cell dysfunction in type 2 diabetes: Implications of inflammation and oxidative stress. World J diabetes. (2023) 14:130–46. doi: 10.4239/wjd.v14.i3.130 PMC10075035 37035220

[50] Wronka M Krzemińska J Młynarska E Rysz J Franczyk B . The influence of lifestyle and treatment on oxidative stress and inflammation in diabetes. Int J Mol Sci. (2022) 23:15743. doi: 10.3390/ijms232415743 36555387 PMC9778895

[51] Zhang X Liu L . Effects of Resveratrol Extract Powder on blood sugar, superoxide dismutase, catalase, glutathione peroxidase and malondialdehyde in patients with type 2 diabetes. Hebei J Traditional Chin Med. (2017) 39:5. doi: 10.3969/j.issn.1002-2619.2017.01.005

[52] Shu L Zhao H Huang W Hou G Song G Ma H . Resveratrol Upregulates mmu-miR-363-3p via the PI3K-Akt Pathway to Improve Insulin Resistance Induced by a High-Fat Diet in Mice. Diabetes Metab syndrome obesity: Targets Ther. (2020) 13:391–403. doi: 10.2147/DMSO.S240956 PMC7027849 32104036

[53] Huang X Sun J Chen G Niu C Wang Y Zhao C . Resveratrol promotes diabetic wound healing via SIRT1-FOXO1-c-myc signaling pathway-mediated angiogenesis. Front Pharmacol. (2019) 10:421. doi: 10.3389/fphar.2019.00421 31068817 PMC6491521

[54] Szkudelska K Deniziak M Hertig I Wojciechowicz T Tyczewska M Jaroszewska M . Effects of resveratrol in goto-kakizaki rat, a model of type 2 diabetes. Nutrients. (2019) 11:2488. doi: 10.3390/nu11102488 31623226 PMC6836277

[55] Luo G Xiao L Wang D Wang N Luo C Yang X . Resveratrol attenuates excessive ethanol exposure-induced β-cell senescence in rats: A critical role for the NAD(+)/SIRT1-p38MAPK/p16 pathway. J Nutr Biochem. (2021) 89:108568. doi: 10.1016/j.jnutbio.2020.108568 33326842

[56] Li H Shen Y Xiao H Sun W . Resveratrol attenuates rotenone-induced inflammation and oxidative stress via STAT1 and Nrf2/Keap1/SLC7A11 pathway in a microglia cell line. Pathology Res practice. (2021) 225:153576. doi: 10.1016/j.prp.2021.153576 34391968

[57] Yang C Luo P Chen SJ . Resveratrol sustains intestinal barrier integrity, improves antioxidant capacity, and alleviates inflammation in the jejunum of ducks exposed to acute heat stress. Poultry science. (2021) 100:101459. doi: 10.1016/j.psj.2021.101459 PMC8498463 34614430

[58] Cichocki M Paluszczak J Szaefer H Piechowiak A Rimando AM Baer-Dubowska W . Pterostilbene is equally potent as resveratrol in inhibiting 12-O-tetradecanoylphorbol-13-acetate activated NFkappaB, AP-1, COX-2, and iNOS in mouse epidermis. Mol Nutr Food Res. (2008) 52 Suppl 1:S62–70. doi: 10.1002/mnfr.200700466 18551458

[59] Yang B Long H Wang Z Zeng D Zhang B Long Q . Resveratrol activates nrf-2 signal to inhibit apoptosis,Oxidative damage and inflammatory response of osteoarthritis chondrocyte induced by H2O2. Chin J Modern Appl Pharmacy. (2021) 38:2359–66. doi: 10.13748/j.cnki.issn1007-7693.2021.19.004

