# Supplementary material for: The efficacy of resveratrol supplementation on inflammation and oxidative stress in type-2 diabetes mellitus patients: randomized double-blind placebo meta-analysis
Source: Front Endocrinol (Lausanne). 2025 Jan 13;15:1463027. doi: 10.3389/fendo.2024.1463027 (PMC11771208; doi:10.3389/fendo.2024.1463027)
Supplement: Supplementary file 1 [file DataSheet1.docx]

**Supplemental Method**

Search strategies for Pubmed, ClinicalTrials, ProQuest Dissertations and Theses ,Web of Science,Embase and the Cochrane Library.

**PubMed: 74 results**

(((((((((((((((((((((((((((((((((Diabetes Mellitus, Type 2[MeSH Terms]) OR (Diabetes Mellitus, Adult-Onset[Title/Abstract])) OR (Adult-Onset Diabetes Mellitus[Title/Abstract])) OR (Diabetes Mellitus, Adult Onset[Title/Abstract])) OR (Diabetes Mellitus, Ketosis-Resistant[Title/Abstract])) OR (Diabetes Mellitus, Ketosis Resistant[Title/Abstract])) OR (Ketosis-Resistant Diabetes Mellitus[Title/Abstract])) OR (Diabetes Mellitus, Non Insulin Dependent[Title/Abstract])) OR (Diabetes Mellitus, Non-Insulin-Dependent[Title/Abstract])) OR (Non-Insulin-Dependent Diabetes Mellitus[Title/Abstract])) OR (Diabetes Mellitus, Stable[Title/Abstract])) OR (Stable Diabetes Mellitus[Title/Abstract])) OR (Diabetes Mellitus, Type II[Title/Abstract])) OR (NIDDM[Title/Abstract])) OR (Diabetes Mellitus, Noninsulin Dependent[Title/Abstract])) OR (Diabetes Mellitus, Maturity-Onset[Title/Abstract])) OR (Diabetes Mellitus, Maturity Onset[Title/Abstract])) OR (Maturity-Onset Diabetes Mellitus[Title/Abstract])) OR (Maturity Onset Diabetes Mellitus[Title/Abstract])) OR (MODY[Title/Abstract])) OR (Diabetes Mellitus, Slow-Onset[Title/Abstract])) OR (Diabetes Mellitus, Slow Onset[Title/Abstract])) OR (Slow-Onset Diabetes Mellitus[Title/Abstract])) OR (Type 2 Diabetes Mellitus[Title/Abstract])) OR (Noninsulin-Dependent Diabetes Mellitus[Title/Abstract])) OR (Noninsulin Dependent Diabetes Mellitus[Title/Abstract])) OR (Maturity-Onset Diabetes[Title/Abstract])) OR (Diabetes, Maturity-Onset[Title/Abstract])) OR (Maturity Onset Diabetes[Title/Abstract])) OR (Type 2 Diabetes[Title/Abstract])) OR (Diabetes, Type 2[Title/Abstract])) OR (Diabetes Mellitus, Noninsulin-Dependent[Title/Abstract])) AND (((((((((((((((((Resveratrol[MeSH Terms])Resvera OR (trol[Title/Abstract])) OR (3,4',5-Stilbenetriol[Title/Abstract])) OR (3,5,4'-Trihydroxystilbene[Title/Abstract])) OR (3,4',5-Trihydroxystilbene[Title/Abstract])) OR (trans-Resveratrol[Title/Abstract])) OR (trans Resveratrol[Title/Abstract])) OR (Resveratrol-3-sulfate[Title/Abstract])) OR (Resveratrol 3 sulfate[Title/Abstract])) OR (SRT 501[Title/Abstract])) OR (SRT-501[Title/Abstract])) OR (SRT501[Title/Abstract])) OR (cis-Resveratrol[Title/Abstract])) OR (cis Resveratrol[Title/Abstract])) OR (Resveratrol, (Z)-[Title/Abstract])) OR (trans-Resveratrol-3-O-sulfate[Title/Abstract])) OR (trans Resveratrol 3 O sulfate[Title/Abstract]))) AND ((((((((Randomized Controlled Trial[Publication Type]) OR (Randomized Controlled Trials as Topic[MeSH Terms])) OR (Randomized Controlled Trials[Title/Abstract])) OR (Randomized Controlled Trial[Title/Abstract])) OR (Clinical Trials, Randomized[Title/Abstract])) OR (Trials, Randomized Clinical[Title/Abstract])) OR (Controlled Clinical Trials, Randomized[Title/Abstract])) OR (Random*[Title/Abstract]))

**Embase: 240 results**

#1：diabetes AND mellitus, AND type AND 2 OR (diabetes AND mellitus, AND 'adult onset') OR ('adult onset' AND diabetes AND mellitus) OR (diabetes AND mellitus, AND adult AND onset) OR (diabetes AND mellitus, AND 'ketosis resistant') OR (diabetes AND mellitus, AND ketosis AND resistant) OR ('ketosis resistant' AND diabetes AND mellitus) OR (diabetes AND mellitus, AND non AND insulin AND dependent) OR 'non insulin dependent diabetes mellitus' OR (diabetes AND mellitus, AND stable) OR (stable AND diabetes AND mellitus) OR (diabetes AND mellitus, AND type AND ii) OR niddm OR (diabetes AND mellitus, AND noninsulin AND dependent) OR (diabetes AND mellitus, AND maturity AND onset) OR (maturity AND onset AND diabetes AND mellitus) OR mody OR (diabetes AND mellitus, AND slow AND onset) OR ('slow onset' AND diabetes AND mellitus) OR (type AND 2 AND diabetes AND mellitus) OR (noninsulin AND dependent AND diabetes AND mellitus) OR ('maturity onset' AND diabetes) OR (diabetes, AND 'maturity onset') OR (maturity AND onset AND diabetes) OR (type AND 2 AND diabetes) OR (diabetes, AND type AND 2) OR (diabetes AND mellitus, AND 'noninsulin dependent')

#2：'resveratrol' OR 'trans resveratrol' OR (trans AND resveratrol) OR 'resveratrol 3 sulfate' OR (resveratrol AND 3 AND sulfate) OR (srt AND 501) OR 'cis resveratrol' OR 'trans resveratrol 3 o sulfate' OR (trans AND resveratrol AND 3 AND o AND sulfate)

#3：'randomized controlled trial' OR (randomized AND controlled AND trials AND as AND topic) OR (randomized AND controlled AND trials) OR (randomized AND controlled AND trial) OR (clinical AND trials, AND randomized) OR (trials, AND randomized AND clinical) OR (controlled AND clinical AND trials, AND randomized) OR random*

#1 AND #2 AND #3

**Cochrane Library: 66 results**

#1 (Diabetes Mellitus, Type 2):ti,ab,kw OR (Diabetes Mellitus, Adult-Onset):ti,ab,kw OR (Adult-Onset Diabetes Mellitus):ti,ab,kw OR (Diabetes Mellitus, Adult Onset):ti,ab,kw OR (Diabetes Mellitus, Ketosis-Resistant):ti,ab,kw (Word variations have been searched) 57139

#2 (Diabetes Mellitus, Ketosis Resistant):ti,ab,kw OR (Ketosis-Resistant Diabetes Mellitus):ti,ab,kw OR (Diabetes Mellitus, Non Insulin Dependent):ti,ab,kw OR (Diabetes Mellitus, Non-Insulin-Dependent):ti,ab,kw OR (Non-Insulin-Dependent Diabetes Mellitus):ti,ab,kw (Word variations have been searched) 24378

#3 (Diabetes Mellitus, Stable):ti,ab,kw OR (Stable Diabetes Mellitus):ti,ab,kw OR (Diabetes Mellitus, Type II):ti,ab,kw OR (NIDDM):ti,ab,kw OR (Diabetes Mellitus, Noninsulin Dependent):ti,ab,kw (Word variations have been searched) 10866

#4 (Diabetes Mellitus, Maturity-Onset):ti,ab,kw OR (Diabetes Mellitus, Maturity Onset):ti,ab,kw OR (Maturity-Onset Diabetes Mellitus):ti,ab,kw OR (Maturity Onset Diabetes Mellitus):ti,ab,kw OR (MODY):ti,ab,kw (Word variations have been searched) 259

#5 (Diabetes Mellitus, Slow-Onset):ti,ab,kw OR (Diabetes Mellitus, Slow Onset):ti,ab,kw OR (Slow-Onset Diabetes Mellitus):ti,ab,kw OR (Type 2 Diabetes Mellitus):ti,ab,kw OR (Noninsulin-Dependent Diabetes Mellitus):ti,ab,kw (Word variations have been searched) 56546

#6 (Noninsulin Dependent Diabetes Mellitus):ti,ab,kw OR (Maturity-Onset Diabetes):ti,ab,kw OR (Diabetes, Maturity-Onset):ti,ab,kw OR (Maturity Onset Diabetes):ti,ab,kw OR (Type 2 Diabetes):ti,ab,kw (Word variations have been searched) 66270

#7 #1 OR #2 OR #3 OR #4 OR #5 OR #6 70707

#8 (Resveratrol):ti,ab,kw OR (trans resveratrol):ti,ab,kw AND (Resveratrol 3 sulfate):ti,ab,kw AND (SRT 501):ti,ab,kw AND (cis Resveratrol):ti,ab,kw (Word variations have been searched) 837

#9 (Randomized Controlled Trial):ti,ab,kw OR (Randomized Controlled Trials as Topic):ti,ab,kw AND (Randomized Controlled Trials):ti,ab,kw AND (Randomized Controlled Trial):ti,ab,kw AND (Clinical Trials, Randomized):ti,ab,kw (Word variations have been searched) 895281

#10 #7 AND #8 AND #9 66

**Web of Science:170 results**

Diabetes Mellitus, Type 2 (Topic) or Diabetes Mellitus, Adult-Onset (Topic) or Adult-Onset Diabetes Mellitus (Topic) or Diabetes Mellitus, Adult Onset (Topic) or Diabetes Mellitus, Ketosis-Resistant (Topic) or Diabetes Mellitus, Ketosis Resistant (Topic) or Ketosis-Resistant Diabetes Mellitus (Topic) or Diabetes Mellitus, Non Insulin Dependent (Topic) or Diabetes Mellitus, Non-Insulin-Dependent (Topic) or Non-Insulin-Dependent Diabetes Mellitus (Topic) or Diabetes Mellitus, Stable (Topic) or Stable Diabetes Mellitus (Topic) or Diabetes Mellitus, Type II (Topic) or NIDDM (Topic) or Diabetes Mellitus, Noninsulin Dependent (Topic) or Diabetes Mellitus, Maturity-Onset (Topic) or Diabetes Mellitus, Maturity Onset (Topic) or Maturity-Onset Diabetes Mellitus (Topic) or Maturity Onset Diabetes Mellitus (Topic) or MODY (Topic) or Diabetes Mellitus, Slow-Onset (Topic) or Diabetes Mellitus, Slow Onset (Topic) or Slow-Onset Diabetes Mellitus (Topic) or Type 2 Diabetes Mellitus (Topic) or Noninsulin-Dependent Diabetes Mellitus (Topic) or Noninsulin Dependent Diabetes Mellitus (Topic) or Maturity-Onset Diabetes (Topic) or Diabetes, Maturity-Onset (Topic) or Maturity Onset Diabetes (Topic) or Type 2 Diabetes (Topic) or Diabetes, Type 2 (Topic) or Diabetes Mellitus, Noninsulin-Dependent (Topic)

Resveratrol (Topic) or 3,4',5-Stilbenetriol (Topic) or 3,5,4'-Trihydroxystilbene (Topic) or 3,4',5-Trihydroxystilbene (Topic) or trans-Resveratrol (Topic) or trans Resveratrol (Topic) or Resveratrol-3-sulfate (Topic) or Resveratrol 3 sulfate (Topic) or SRT 501 (Topic) or SRT-501 (Topic) or SRT501 (Topic) or cis-Resveratrol (Topic) or cis Resveratrol (Topic) or Resveratrol, (Z)- (Topic) or trans-Resveratrol-3-O-sulfate (Topic) or trans Resveratrol 3 O sulfate (Topic)

Randomized Controlled Trial (Topic) or Randomized Controlled Trials as Topic (Topic) or Randomized Controlled Trials (Topic) or Randomized Controlled Trial (Topic) or Clinical Trials, Randomized (Topic) or Trials, Randomized Clinical (Topic) or Controlled Clinical Trials, Randomized (Topic) or Random* (Topic).

**ClinicalTrials:25 results**

(diabetes AND mellitus, AND type AND 2 OR (diabetes AND mellitus, AND 'adult onset') OR ('adult onset' AND diabetes AND mellitus) OR (diabetes AND mellitus, AND adult AND onset) OR (diabetes AND mellitus, AND 'ketosis resistant') OR (diabetes AND mellitus, AND ketosis AND resistant) OR ('ketosis resistant' AND diabetes AND mellitus) OR (diabetes AND mellitus, AND non AND insulin AND dependent) OR 'non insulin dependent diabetes mellitus' OR (diabetes AND mellitus, AND stable) OR (stable AND diabetes AND mellitus) OR (diabetes AND mellitus, AND type AND ii) OR niddm OR (diabetes AND mellitus, AND noninsulin AND dependent) OR (diabetes AND mellitus, AND maturity AND onset) OR (maturity AND onset AND diabetes AND mellitus) OR mody OR (diabetes AND mellitus, AND slow AND onset) OR ('slow onset' AND diabetes AND mellitus) OR (type AND 2 AND diabetes AND mellitus) OR (noninsulin AND dependent AND diabetes AND mellitus) OR ('maturity onset' AND diabetes) OR (diabetes, AND 'maturity onset') OR (maturity AND onset AND diabetes) OR (type AND 2 AND diabetes) OR (diabetes, AND type AND 2) OR (diabetes AND mellitus, AND 'noninsulin dependent')) AND ('resveratrol' OR 'trans resveratrol' OR (trans AND resveratrol) OR 'resveratrol 3 sulfate' OR (resveratrol AND 3 AND sulfate) OR (srt AND 501) OR 'cis resveratrol' OR 'trans resveratrol 3 o sulfate' OR (trans AND resveratrol AND 3 AND o AND sulfate))

**ProQuest Dissertations and Theses: 3 results**

noft(Diabetes Mellitus, Type 2) OR noft(Diabetes Mellitus, Adult-Onset) OR noft(Adult-Onset Diabetes Mellitus) OR noft(Diabetes Mellitus, Ketosis-Resistant) OR noft(Ketosis-Resistant Diabetes Mellitus) OR noft(Diabetes Mellitus, Non Insulin Dependent) OR noft(Non-Insulin-Dependent Diabetes Mellitus) OR noft(Diabetes Mellitus, Stable) OR noft(Diabetes Mellitus, Type II) OR noft(NIDDM) OR noft(Diabetes Mellitus, Noninsulin Dependent) OR noft(Diabetes Mellitus, Maturity-Onset) OR noft(Maturity Onset Diabetes Mellitus) OR noft(MODY) OR noft(Diabetes Mellitus, Slow Onset) OR noft(Slow-Onset Diabetes Mellitus) OR noft(Type 2 Diabetes Mellitus) OR noft(Noninsulin-Dependent Diabetes Mellitus) OR noft(Noninsulin Dependent Diabetes Mellitus) OR noft(Maturity Onset Diabetes) OR noft(Type 2 Diabetes) OR noft(Diabetes, Type 2) OR noft(Diabetes Mellitus, Noninsulin-Dependent)

AND

noft(Resveratrol) OR noft(3,4',5-Stilbenetriol) OR noft(3,5,4'-Trihydroxystilbene) OR noft(3,4',5-Trihydroxystilbene) OR noft(trans-Resveratrol) OR noft(trans Resveratrol) OR noft(Resveratrol-3-sulfate) OR noft(Resveratrol 3 sulfate) OR noft(SRT 501) OR noft(SRT-501) OR noft(SRT501) OR noft(cis-Resveratrol) OR noft(cis Resveratrol) OR noft(Resveratrol, (Z)-) OR noft(trans-Resveratrol-3-O-sulfate) OR noft(trans Resveratrol 3 O sulfate) OR SRT501 OR cis-Resveratrol OR (cis Resveratrol) OR (Resveratrol, (Z) -) OR trans-Resveratrol-3-O-sulfate OR (trans Resveratrol 3 O sulfate)

AND

(Randomized Controlled Trial) OR (Randomized Controlled Trials as Topic) OR (Randomized Controlled Trials) OR (Randomized Controlled Trial) OR (Clinical Trials, Randomized) OR (Trials, Randomized Clinical) OR (Controlled Clinical Trials, Randomized) OR Random*
